# Framework for Microdosing Odors in Virtual Reality for Psychophysiological Stress Training

**DOI:** 10.3390/s24217046

**Published:** 2024-10-31

**Authors:** Daniel Anheuer, Brid Karacan, Lara Herzog, Nora Weigel, Silja Meyer-Nieberg, Thomas Gebhardt, Jessica Freiherr, Martin Richter, Armin Leopold, Monika Eder, Marko Hofmann, Karl-Heinz Renner, Cornelia Küsel

**Affiliations:** 1 Fraunhofer Institute for Electronic Microsystems and Solid State Technologies, 80686 Munich, Germany; 2 Sensory Analytics and Technologies, Fraunhofer Institute for Process Engineering and Packaging IVV, 85354 Freising, Germany; 3 Department of Computer Science, University of the Bundeswehr Munich, 85579 Neubiberg, Germany; 4 Department of Psychiatry and Psychotherapy, Friedrich-Alexander-Universität Erlangen-Nürnberg, 91054 Erlangen, Germany; 5 Department of Human Sciences, University of the Bundeswehr Munich, 85579 Neubiberg, Germany

**Keywords:** stress training, olfaction, odor actuator, physiological response, psychological response, personalized odor presentation, micropump, scent dosing, olfactory display, multisensory VR

## Abstract

To better cope with stress in emergencies, emergency personnel undergo virtual reality (VR) stress training. Such training typically includes visual, auditory and sometimes tactile impressions, whereas olfactory stimuli are mostly neglected. This concept paper therefore examines whether odors might be beneficial for further enhancing the experience of presence and immersion into a simulated environment. The aim is to demonstrate the benefits of VR civilian stress training for emergency personnel and to investigate the role of odors as stressors by manipulating the degree of perceived psychophysiological stress via olfactory impressions. Moreover, the current paper presents the development and validation of a convenient and portable fragrance dosing system that allows personalized odor presentation in VR. The presented system can transport reproducible small quantities of an air-fragrance mixture close to the human nose using piezoelectric stainless steel micropumps. The results of the fluidic system validation indicate that the micropump is suitable for releasing odors close to the nose with constant amounts of odor presentation. Furthermore, the theoretical background and the planned experimental design of VR stress training, including odor presentation via olfactory VR technology, are elucidated.

## 1. Introduction

Virtual reality (VR) enables people to immerse themselves in simulated worlds. It offers users within a 360° perspective the feeling of presence and the possibility to freely discover and interact with a virtual environment. VR environments typically include the presentation of visual, auditory and sometimes tactile impressions, whereas olfactory stimuli are mostly neglected [1,2]. In fact, odors might be particularly suitable for further enhancing the experience of presence and immersion into a simulated scene. In other words, the absence of sensory impressions when they are normally expected, e.g., the smell of fire while seeing a fire, might decrease perceived realism in a simulated environment [3,4,5].

In recent years, a variety of different concepts and prototypes have been developed in the scientific and corporate sectors for repeatable and portable odor delivery, e.g., using micropumps, evaporation of liquid solutions with heating elements, or the use of atomizers. However, these existing systems are still in development and mostly unavailable on the global market due to various challenges in odor presentation, e.g., the optimization of the dosing process, release rate and localization and duration of odor stimulation [1,6,7].

### 1.1. Main Aim of the Work

The aim of this conceptual paper is to illuminate the benefits of VR stress training for civilian emergency personnel and to investigate the role of odors as stressors by manipulating the degree of perceived psychophysiological stress via olfactory impressions. Moreover, the current paper will present the development and validation of a convenient and portable fragrance dosing system that allows personalized odor presentation in VR. The system can transport reproducible small quantities of an air-fragrance mixture close to the human nasal passage using piezoelectric stainless steel micropumps. We are thus addressing the research gap regarding the use of personalized odor presentation in VR for this specific application. To this end, we developed and validated the fragrance dosing system and identified relevant odors as stressors.

Within this paper, we present the current scientific state on olfactory virtual reality technology and odor dosing. To better understand the contextual framework, we explain below our approach to using odors in VR stress training for emergency personnel with the current state of science of odors in stress training, the overall concept of psychophysiological stress measurement in VR, and the psychological and physiological effects of odors. Our conceptual paper enables the scientific exchange of different research groups involved in developing virtual stress training scenarios. As a result, this promotes a standardized experimental approach and thus supports the comparability of future studies.

### 1.2. Olfactory Virtual Reality Technology

There are few cost-effective odor applications available for a constant, replicable release of olfactory stimuli in VR (for an overview, see Howell et al. [1]). For instance, Howell et al. [1] developed a reproducible but immobile olfactory display to present odors in VR environments that could be replicated cost-effectively and conducted a study on both positive and negative observed effects on user experience. A non-intrusive and low-cost olfactory display with one odor for VR glasses is shown by Guimarães et al. [8]. However, no valves are used in this system with only one odor channel, making it difficult to achieve timely and precise odor application. Liu et al. [9] presented a conceptual soft miniaturized and wireless-connected olfactory interface for VR applications in 2022. Fragrance-enriched and heated paraffin waxes were used, whereby the response time is slow and remaining scent molecules are expected to remain perceptible for an extended period after actuation. Niedenthal et al. [10] developed and validated an olfactory display fitted in a VR hand controller. The compact and low-cost system with four scent channels creates an airflow through a ventilator controlled by active valves and enables objects in VR to be smelled when placed close to the nose. Further miniaturization of the system could be problematic due to the components used. In addition, the air mixer could cause unwanted mixing of odors.

Companies such as OVR Technology and Aromajoin Corporation hold several patents and are working on portable fragrance dispensing systems. Since the market launch of these concepts is pending, their functionality is still unclear. One reason for the absence of efficient, lightweight and portable olfactory delivery systems for VR systems on the market is the challenging requirements of enabling an immersive user experience. The objective of administering odors near the nose is to provide odor scenarios for a single person within one individual breath. A combination of multiple scents corresponds to an odor scenario similar to a picture scenario (movie) or sound scenario (music) [11]. Tewell and Ranasinghe provide a recent review of a multitude of olfactory display designs for virtual reality with a focus on Head-Mounted Displays (HMD) and Cave Automatic Virtual Environment (CAVE) [7]. They present a classification based on three categories: delivery method (like airflow, heat and atomization), presentation approaches (ubiquitous, wearable, handheld), and application area (e.g., health, perception, engineering, gaming, education) [7].

When presenting different sensory impressions simultaneously, not only the congruence between the impressions must be taken into account, but also the time window in which they are presented. For example, Murray et al. [12] identified a time window of 5–15 s before/after the congruent visual stimuli as acceptable. Another challenge of adding odor to VR is the adaptation effect [7], which describes the habituation of the olfactory system to persistent olfactory stimuli, wherein perception is reduced or the odors are no longer perceived at all. To counteract this effect, an event-related design is often used in experiments. Here, odor stimuli are only presented for a few seconds, followed by a change in odor quality or a pause (presenting neutral air) until the next odor presentation.

### 1.3. Odor Dosing with Piezoelectric Metal Micropumps

The use of micropumps enable gas delivery systems with high dosing accuracy and reproducibility, both key aspects for odor applications. A wide variety of micropumps exist and are built with different materials such as metal [13] silicon [11] or material combinations of metal, silicon and polymer [14]. Actuation mechanisms of micropumps vary greatly from piezoelectric [13] and electrostatic [15] to electromagnetically driven pumps [16] and many more. Nevertheless, piezoelectric micropumps show superior performance in multiple aspects, such as pressure generation, counter pressure and flow rate.

**Figure 1:**
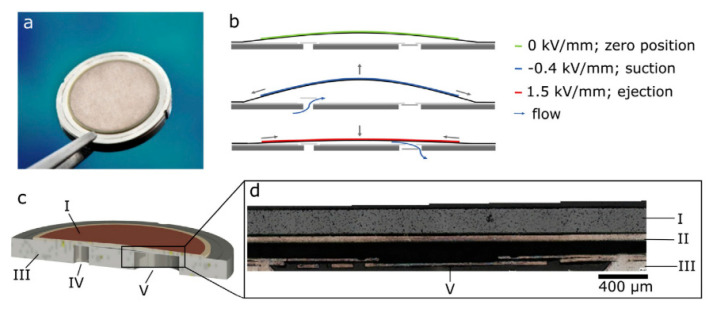
(**a**) Piezoelectric stainless steel micropump P32009 with diameter of 20 mm and height of 1.5 mm; (**b**) working principle of the micropump with a piezoelectric actuator and two passive check valves; (**c**) pump model cross-section with piezoelectric actuator (I), pump body (III), fluid inlet (IV) and fluid outlet (V); (**d**) microsection of the fluid outlet valve with actuator diaphragm (II) [13].

To dose precise amounts of odor-enriched gases, piezoelectric stainless steel micropumps with passive flap valves, as depicted in Figure 1, are used in the current study [13]. Gas flow rates of 60–70 mL/min air with a sinusoidal actuation signal between $U_{-}=-80$ V and $U_{+}=300$ V at frequencies of f=200 Hz without counterpressure are generated with this pump model.

The advantages of using micropumps for dosing odorants are explained in detail in the following concerning the requirements of an odor dosing system. These micropumps are designed to pump odor-enriched gases from a saturated headspace, which is further detailed in Section 2.2. Important properties of scent dosing systems are the high reproducibility of the dosed gas volume and the possibility of dosing various quantities continuously over time. The small size of the micropumps enables the minimization of dead volumes to create a minimal time difference between the activation of the pump and the olfactory stimulus. Parallelization of micropumps enables the use of several odorants in a compact setup and prevents cross-contamination, as each pump is provided with a separate odor channel. Another important factor is the switching time between odorants and the response time. By reducing the dead volume between the odor pumps and the human nose, it is possible to reduce these delays. The disadvantage is the necessary proximity of the pump and odor reservoir to the nose, as possible leakages of the system, which can always occur, cause disturbing emissions and reduce the user experience. The system with four micropumps can be integrated into a VR headset and is therefore mobile and allows for control and activation directly from the virtual environment. Furthermore, as many commercially available components as possible shall be used for the dosing system to manufacture it in a cost-effective and reproducible process.

### 1.4. Use of Odors in VR Stress Training for Emergency Personnel

Emergency personnel face many challenges in daily work. However, although they are often confronted with traumatic events, most of them do not develop any serious psychological problems [17]. One reason for this might be a strong resilience [17]. Our research group has developed concepts for stress training that address (1) mission-specific stressors as well as (2) organizationally relevant stressors [18]. Mission-specific stressors include visual stressors like different injuries with varying degrees of severity (gradual increase), the number of injured persons (gradual increase), confusion of the scenario, different weather conditions and formation of smoke (primary stressor) (gradual increase in deterioration of visibility). Additionally, it addresses auditory stressors (e.g., cries of pain, mixed voices, radio traffic and examination noises on the patient) and olfactory stressors. In particular, primordial stressors such as smoke and fire are particularly suitable for converting odors into VR, but patient-related odors like sweat, blood and vomit are possibly relevant olfactory stressors. Organizational relevant stressors are, e.g., related to working conditions, the specific understanding of roles, and the resulting conflicts.

To build up a strong resilience, specific and recurring training is required that prepares the emergency personnel for their everyday work in a holistic way, using as many senses as possible. The use of odors in VR stress training for emergency personnel is a relatively new field of research. However, according to field experts, odors play a special role in rescue operations and should therefore be integrated into stress training. Older studies, such as those by Marmar et al. [19] or Raphael et al. [20], already point to a possible role of odors as a stressor in the context of studies on stressors for emergency personnel. A research report from 2005 already dealt with the effect of odors on military personnel in the context of the Gulf War syndrome [21]. The authors present two main results. Firstly, odors that are associated with a stressful situation can subsequently trigger negative reactions. Secondly, negative reactions can be inhibited if the odor can be experienced beforehand in a non-stressful and controlled context. The gradual increase in odors and the possibility of using VR were not investigated in this study.

There are several advantages of using VR as a training scenario (e.g., for the fire brigade or ambulance staff) in comparison to real-life training [2,22]. Immersive 360° videos allow for a fully controlled and influenceable environment. Users can interrupt and resume the training at any time without any harm. The degree of presence/perceptual realism of the virtual environment can be increased gradually depending on individual emotional resilience. For instance, immersion into the simulated world can be gradually increased by adding successively congruent sensory impressions (visual, auditory, tactile, olfactory) [3]. Already Dinh et al. [23] examined the impact of multisensory stimuli (visual, auditory, tactile and olfactory) in virtual environments, indicating a greater sense of presence by adding an odor. Moreover, Ghinea and Ademoye [24] showed that watching video clips while presenting a congruent odor (vs. an odor neutralizer) increased the sense of reality, and Archer et al. [25] were able to show that odors in VR enhance the experience of presence in contrast to VR without odors. However, the use of odors did not change the emotional state, and a physiological reaction to the odor was only shown when the odor was new and not on the second playthrough. Thus, further research is needed to elucidate the significance of odors for emotional and physiological changes.

### 1.5. Triggering Psychophysiological Stress via Odors in VR

Psychological stress manifests physiologically in humans, among others, through two main pathways: the activation of the sympathetic–adrenal–medullary (SAM) system and the slower-acting activation of the hypothalamic–pituitary–adrenal (HPA) axis. This activation triggers the release of hormones, increasing heart rate, blood pressure, and blood glucose levels, preparing the body for immediate action [26]. This activation also includes the autonomic nervous systems (ANS). Changes in the heart rate variability, the skin conductance response or cortisol level, among others, can be measured to quantify the effects of stress-related physiological activation [27]. The effect on each measurable physiological parameter depends on each individual’s own experience and thus its reaction to as well as the severity of the respective stressor posed in virtual reality [28].

Physiological responses to stress can be measured using different technical systems, among others, such as electroencephalography for detecting brain activity, skin conductance response to evaluate electrodermal activity, sensors for respiration, as well as electromyography for detecting both voluntary and involuntary muscle movement [29]. Electrocardiography (ECG) and photoplethysmography (PPG) are among the most common methods used to obtain the cardiovascular signals required to calculate heart rate and heart rate variability metrics [30]. However, both methods suffer from artifact and noise corruption due to their approach to measurement, which can affect the subsequently computed results substantially [30]. Noise and artifact distortion is not limited to ECG and PPG, but the other technical measurement systems suffer from similar system-specific challenges as well. Thus, the data preprocessing of collected physiological signals is crucial to obtaining reliable results for further interpretation [30]. In addition, it should be mentioned that many physiological parameters are not only influenced by the human stress response but also by other biophysiological processes.

The physiological responses in VR environments are a subject of current research. For instance, they can be measured by using heart rate variability [29] or salivary markers, which comprise, among others, α-amylase, cortisol, or secretory immunoglobulin-A (IgA) [31]. These are obtained at several time points before and after the experiment. The changes in these parameters can be used to quantify the stress response. However, some pitfalls remain since the response depends on several factors, e.g., age. The time delay until a response in the saliva can be measured is significant and prohibits its use whenever a nearly real-time estimate of the subject‘s state is required. Stress detection, therefore, typically focuses on physiological parameters that do not exhibit such a long response time. It needs to be distinguished from a subsequent statistical analysis that aims to investigate, among others, whether significant differences in the data occurred during the experiment. Instead, the objective is to derive a statistical or machine learning model that provides a reliable state estimate. Most algorithms used in this context belong to the field of supervised learning, where labels are required for all input data [32]. The establishment of stress ground truth may lie in a combination of both subjective and objective methods of stress assessment [29]. Some physiological parameters cannot be used for online stress detection, but machine learning can be used to overcome the problems. Most algorithms used in this context belong to the field of supervised learning, where labels are required for all input data [32]. Despite the challenges, the advantage of physiological stress measures is the provision of quantitative indicators related to the body’s stress response without the subjective influence of the respective person, such as the response bias using questionnaires for self-assessment [29]. This is the case for the physiological variables that are not consciously controllable by the participant. However, the establishment of stress ground truth may lie in a combination of both subjective and objective methods of stress assessment [29].

VR itself also presents difficulties. VR motion sickness can lead to the activation of stress-related physiological changes [33]. Assuming the motion sickness itself is not part of the stressors, the differentiation between its influence and the stress induced by the actual stressors on the physiological parameters is one of the challenges when using objective stress measurement with virtual reality [33].

Evolutionarily, odors had an important function as a warning signal against hazardous, life-threatening situations, such as undiscovered gas leaks, fire or spoiled food [34]. From a neurological point of view, odor stimuli are processed in the limbic system, a brain region that also processes emotions and memories: the amygdala plays an important role in emotional processing, whereas the hippocampus is particularly important for olfactory memory and is sensitive to stress. Thus, odors unintentionally and unconsciously evoke emotional memories. Moreover, the hippocampus regulates the physiology of the body in relation to environmental stimuli together with the amygdala [35]. Therefore, besides emotions and memories, odors may also influence physiological responses [25].

There are still many unanswered questions and contradictory results regarding physiological responses to odors [36]. For instance, Höferl et al. [37] showed that physiological parameters might respond different to various odors. In their study, linalool reduced salivary cortisol levels, whereas it increased blood pressure and heart rate. Springer et al. [38] investigated the effect of the odor of Myrothamnus flabellifolia before and after triggering stress by utilizing an emotion questionnaire, the analysis of saliva samples to determine the concentration of the hormones cortisol and α-amylase and a mobile EEG measurement (to quantify the alpha brain waves). The odor was found to significantly reduce stress. Participants reported significantly fewer negative emotions and showed significantly lower cortisol levels and a trend toward a significant increase in alpha activity compared to the placebo application.

## 2. Materials and Methods

Integrating odors into VR can significantly enhance user experiences in use cases such as training and therapy (e.g., augmented exposure therapy for PTSD; [35]). The schematic dosing concept of the developed odor dosing system as well as the integration into two VR applications are depicted and outlined in the following. By addressing these factors, we aim to advance the development of immersive and realistic olfactory experiences in virtual environments.

### 2.1. Setting

Three VR applications have been developed for an analysis of the effects of odors. The first scenario is a VR stress training for emergency personnel, in which the participants see a mass casualty incident after a car accident on the highway, and participants must provide medical care to the virtual patients (smelling unpleasant odors). The second VR scenario represents a pedestrian zone with market stalls by a lake on a summer’s day (smelling pleasant odors). The third scenario uses the environment of the second scenario but shows a truck crash and shooting in the pedestrian zone (smelling unpleasant odors). The second and third scenarios are developed for the training of emergency commanders.

All VR training was developed with the game engine Unreal 5 to achieve a high degree of immersion using remote rendering for high-quality scenarios. Via Wi-Fi 5 or Wi-Fi 6E, the workstations with the latest graphic processing units stream the content directly to the glasses mentioned below.

Scenario 1 includes a comprehensive scenario of a pile-up on a European highway: multiple cars, trucks, and dozens of differently injured patients. To increase the effect of immersion, the visual, auditive, and olfactory effects are on a maximal level of realism. We focus mainly on highly realistic visualization of wounds, adequate patient emotions, authentic sound and voice effects, and various odors true to reality. With the help of a comprehensive scenario editor, the setup of the auditive effects, all kinds and locations of patients’ traumata, and locations and types of odors can be predefined. To ensure the subject can perceive all effects, we want each injured person to be treated according to the TRIAGE m-START algorithm, which takes about 60 to 90 s. The time limit for our tests is set to 10 min but can be adjusted. While moving closer to the injured person or virtual assets, the intensity of the predefined odors increases and decreases when moving away.

Scenario 3 is focused on a terror disaster with multiple injured persons. The authenticity of such tragic incidents was maximized with the help of subject matter experts’ knowledge and the review of historical events. The visualization of the variety of different traumas due to a truck crash and the shooting in a pedestrian zone is a challenge. To quickly realize adequate speech and audio effects, the AI tool elevenlabs.io was used. Additionally, the basic animations, motions, and facial emotions of the patients are on a very authentic level to increase the feeling of presence and immersion. This is realized with the beta version of the omniverse Audio2Face (https://build.nvidia.com/nvidia/audio2face, accessed on 1 September 2024), which creates expressive facial animations from a single audio source with generative AI. In this scenario, it is also possible to experience a relaxing environment (to test pleasant odors) in which all stressors are deactivated. It includes a relaxing pedestrian zone with market stalls by a lake with soothing sounds.

**Figure 2:**
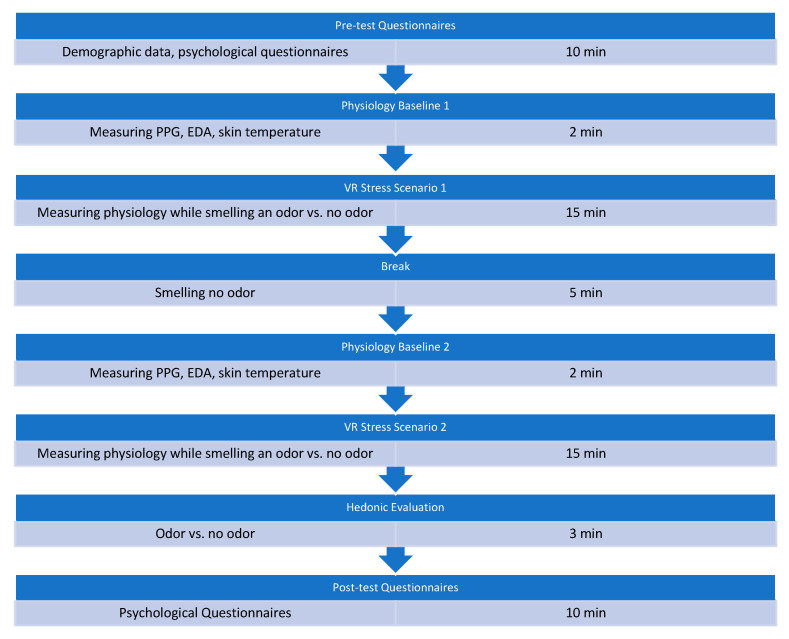
Experimental design.

All scenarios are single-player and might be further developed with multi-user VR settings to allow two people to perceive different odors at the same time. They can be used with a virtual teleportation movement technique or in large physical areas without teleportation. Scenario 1 uses no full-body tracking in contrast to scenarios 2 and 3, which include “Oculus full-body tracking”. At this point, scenarios 2 and 3 use hand-tracking without any controller or tracking device. All scenarios use an HMD, scenario 1 uses HTC VIVE Focus 3 (HTC Corporation, Taoyuan, Taiwan), whereas scenarios 2 and 3 use Meta Quest 3 (128 GB Model S3A, Meta Platforms, Inc., Menlo Park, CA, USA). The odors are presented via the developed micropump, which is installed on the HMD. The wearing comfort was described by the test subjects as not disturbing and lightweight. The experimental design is depicted in Figure 2.

The study starts with a survey of demographic data and psychological questionnaires (see Section 2.4). This is followed by a baseline measurement of physiology (2 min), measuring PPG, SCR and skin temperature. After that, the VR Stress Scenario 1 starts, in which event-related odors (vs. no odor) are released (e.g., seeing fire: smelling fire) and physiological reactions are continuously recorded for 15 min. This is followed by a 5 min break in which participants neither see a VR scenario nor smell odors. Then another baseline measurement follows before VR Stress Scenario 2 starts with the same procedure as in Scenario 1. This is followed by the hedonic evaluation of the odors. Finally, a post-test questionnaire is completed.

Ethical aspects of the use of odors are also considered. A gradual increase in odors and the associated adaptation to the participants are necessary to avoid excessive psychological stress. Unpleasant odors must be used carefully to avoid side effects. Therefore, odor presentations are selected below the concentrations that are usually present in real situations, but above the perception threshold (e.g., for the smell of vomit, fire, etc.).

### 2.2. Micropump Odor Delivery System

In this subsection, the structure of the system is described in detail regarding fluidics and design. First, the dosing concept is explained schematically using a fragrance channel consisting of a piezoelectric micropump, passive non-return valves, Teflon (PTFE) tubing and an odor reservoir. Second, the design of the system is explained and illustrated.

**Figure 3:**
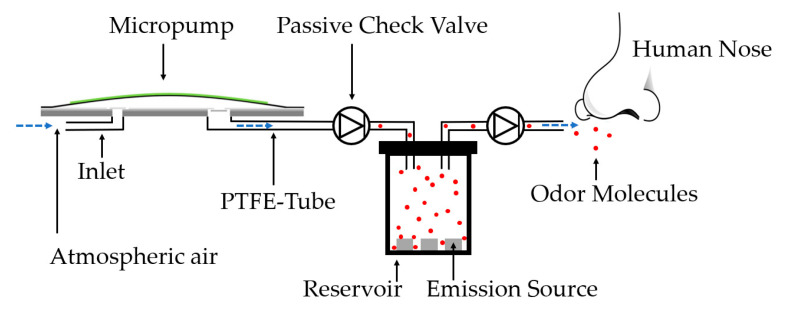
Schematic cross-section of one odor channel consisting of a piezoelectric stainless steel micropump, PTFE-tubing, an odor reservoir with an odor emission source and two passive check-valves. Blue arrows indicate the fluidic path from the atmospheric air inlet to the fluid outlet with odor-enriched air at a close distance to the human nose. Red dots represent odor molecules.

Figure 3 illustrates a schematic cross-section of the system design of one odor channel, which is intended to fulfill the requirements mentioned in Section 1. A piezoelectrically actuated micropump takes in air from the atmosphere and feeds it through a PTFE tube through the first passive check valve (B. Braun Infuvalve [39]). This valve protects the pump from contamination of odorants from the odorant reservoir, allowing the reservoirs to be replaced easily without changing the micropump. After the volume flow passes the first valve, it enters the odor reservoir and accumulates with odorant from the saturated headspace. The reservoir contains an emission source—in this case, a porous cotton pad saturated with fragrance. Subsequently, the enriched air travels through the second check valve, which is supposed to prevent an unwanted leakage rate from the fragrance reservoir toward the human nose. This system is characterized fluidically in Section 3 to verify its compliance with the system requirements.

### 2.3. Measuring Physiological Responses

To measure the physiological response, the experiments will use several established sensor platforms ranging from Shimmer sensors (Shimmer Research Ltd., Dublin, Ireland) (PPG, ECG, SCR) over the Polar ECG (Polar Electro Oy, Kempele, Finland), chest belt H10 to the Empatica E4 (Empatica Inc., Cambridge, MA, USA) (PPG, SCR, skin temperature). The objective is to allow a comparison between several sensor modalities and sensor placements. As stated, measurements in VR are prone to noise and artifacts. In addition, some of the preferred sensor placements, e.g., the insides of hands or feet for electrodermal activity measurements, are not possible. This is the reason why we follow a multimodal and multisensor approach. In the case of SCR, we resort to a shoulder placement of the GSR electrodes, additionally using the often-employed Empatica E4 device on the wrist. As stated before, due to the experimental conditions, the signal processing pipeline must start with data cleaning (noise removal, artifact detection and removal). The methods employed depend strongly on the experimental condition, the signal, and the sensor and may include, for instance, detrending and several filtering steps. The further processing phases depend again on the modality. For example, in the case of electrodermal activity, the signal is typically decomposed into the phasic (fast) and tonic (slow) components (see, among others, [40,41]). Many studies focus on the phasic component, also referred to as the skin conductance response [42]. In the case that a participant reacts to a stimulus, the response can be observed a few seconds later by the time series showing defined transient peaks [42]. For an analysis, among others, the number of peaks, the maximum, or the area under the curve are of interest [42]. To decompose the GSR signal, we will use the established open-source solutions, e.g., NeuroKit2 (0.2.0) [43], which also allows for an event-related analysis, analyzing the data before and after a stimulus presentation.

### 2.4. Measuring Psychological Responses

To assess stress from a psychological point of view, various questionnaires are used before and after the application of the odors. These include pre-tests: the Big-5 Inventory (BFI), the Perceived Stress Questionnaire (PSQ, German Version), Dark Personality (SD3—German Version), Perceived Stress Scale (PSS-10, German Version), Positive and Negative Affect Schedule PANAS (GESIS Panel, German Version); post-tests: the NASA TLX Questionnaire; and for comparison between pre- and post-tests: the Positive and Negative Affect Schedule PANAS (GESIS Panel, German Version).

Data analyses will be performed using SPSS Statistics (Version 29.0.2.0 (20))(IBM SPSS Statistics, Armonk, NY, USA). Depending on the selected study design (within- vs. between-subject) (repeated-measures), ANOVAs and T-tests will be used, e.g., to investigate the main effect of odor presentation on affect. Further, Pearson correlation tests will be used to investigate the relations between odor presentation and psychological and physiological responses.

## 3. Results

This section explores the technology behind the developed odor delivery system, focusing on piezoelectric micropumps as key components. These micropumps, utilizing the piezoelectric effect, provide precise and responsive odor emission, crucial for synchronizing scents with visual and auditory stimuli in VR. The performance of these micropumps in relation to the key requirements for odor delivery is examined, including response time, intensity control, odor switching capabilities and gas flow rates. Furthermore, results of the integration of piezoelectric micropumps for odor delivery onto a head-mounted device (HMD) are detailed.

### 3.1. Micropump Characterization

**Figure 4:**
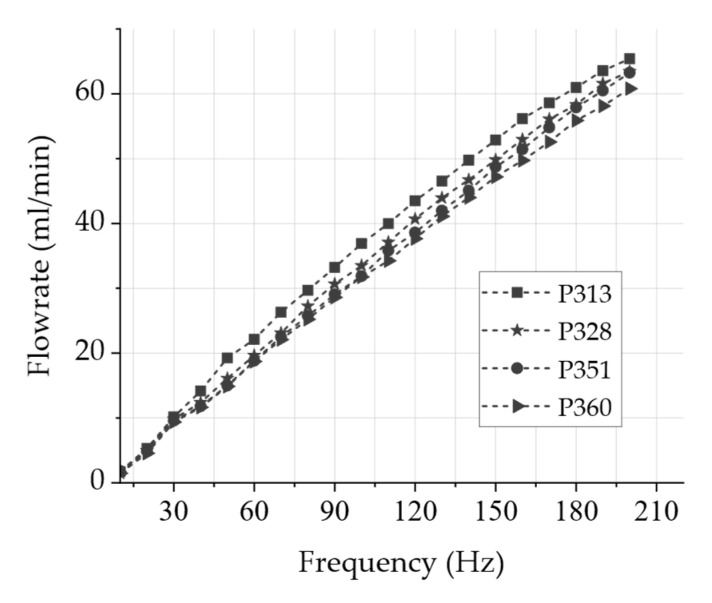
Air volume flow of the four steel micropumps (type P32009) used, with multiple frequencies measured with a Bronkhorst mass flow sensor. A rectangular actuation signal with voltages between $U_{-}=-80$ V and $U_{+}=300$ V using a frequency generator with a high-voltage amplifier was used.

Figure 4 depicts the measured air volume flow of the four steel micropumps (type P32009) used [13]. This measurement is intended to initially determine the flow rate discrepancy between different micropumps to ensure that a calibration for high-dosing repeatability with several channels is achieved during operation. The samples are enclosed the same way in a housing that is used in the overall system. This characteristic frequency-dependent flow rate is measured with a Bronkhorst mass flow sensor.

A rectangular actuation signal with voltages between $U_{-}=-80$ V and $U_{+}=300$ V using a frequency generator with a high-voltage amplifier was used. All micropumps show an almost linear behavior of the flow rate with increasing frequency and deviate from each other with a maximum of approximately 18% from the mean flow rate. However, this deviation can be almost eliminated by a software calibration in the VR scenario. In the overall system, this frequency-dependent flow rate is used to adapt the odor intensity.

### 3.2. Passive Check Valve Characterization

**Figure 5:**
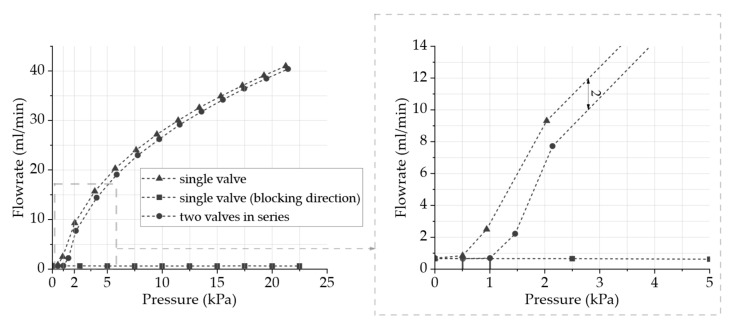
Air volume flow of B. Braun Infuvalve [39] as a function of pressure in the opening and the blocking direction of a single valve and in the opening direction of two valves in series.

Figure 5 depicts measurements of the air volume flow from B. Braun’s Infuvalve [39] passive check valves. In the odor delivery system, two of these valves are used for each channel, as illustrated in Figure 3. Since these commercially available valves are intended for use with liquids, their suitability for this odor-dosing system with gases requires further fluidic evaluation. A low flow resistance and a small opening pressure in the flow direction as well as a minimal leakage rate in the blocking direction are ideal for the application.

The gas flow rate is measured with one single valve as well as two similar valves coupled in series in flow direction and with one single valve in blocking direction with different inlet pressures. The leakage rate of one single valve in the blocking direction indicates a constant leakage volume flow across all applied pressures. It is, therefore, evaluated as a sensor offset without relevance for further evaluation. The opening pressure of a single valve is approximately 1 kPa, and with two valves in series, the opening pressure is around 1.5 Pa, resulting in gas flow rates of approx. 2–3 mL/min. With increasing pressures, the flow rate continues to increase with a decreasing gradient. The measurement shows that the behavior of one valve compared to two valves in series is almost identical and only shifted by the higher opening pressure on the x-axis.

### 3.3. Micropump Odor Delivery System Characterization

**Figure 6:**
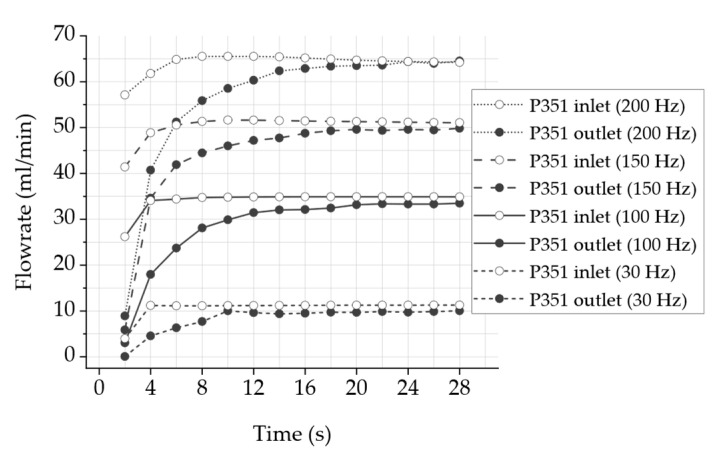
Time-dependent air volume flow as a function of time with micropump P351 and mounted periphery (Figure 3) measured with multiple frequencies, rectangular actuation signal and voltages between $U_{-}=-80$ V and $U_{+}=300$ V compared between inlet and outlet.

Figure 6 plots the continuous air flow rate of the stainless steel micropump P351 of type P32009 at different frequencies over time at the inlet and outlet. This measurement is intended to determine whether the flow rate fluctuates or remains constant for more than 20 s, as a continuous flow rate is an important characteristic of a consistent odor impression. Furthermore, the response time should be as fast as possible to keep the time delay between pumping and scent experience as short as possible. The micropump was operated in the laboratory with a frequency generator and a voltage amplifier with a rectangular signal and voltages between $U_{-}=-80$ V and $U_{+}=300$ V. Thereby, the entire channel with the odorant reservoir is connected to the two passive check valves to reproduce a scenario that is as close to the real system as possible. The flow rate remains almost constant at all frequencies over the measured period, starting at approximately 14 s. Two measurements are displayed for each frequency at the inlet and outlet of the odor channel to quantify the response time. The flow rate at the inlet of the odor channel is close to its maximum value after around 4 s at all frequencies. At the outlet of the channel, the flow rate increases slower and reaches the maximum flow rate after 10–20 s; however, at all frequencies except 200 Hz, the air flow rate remains slightly below the inlet of the channel. These findings must be considered in the design and calibration of the overall system.

**Figure 7:**
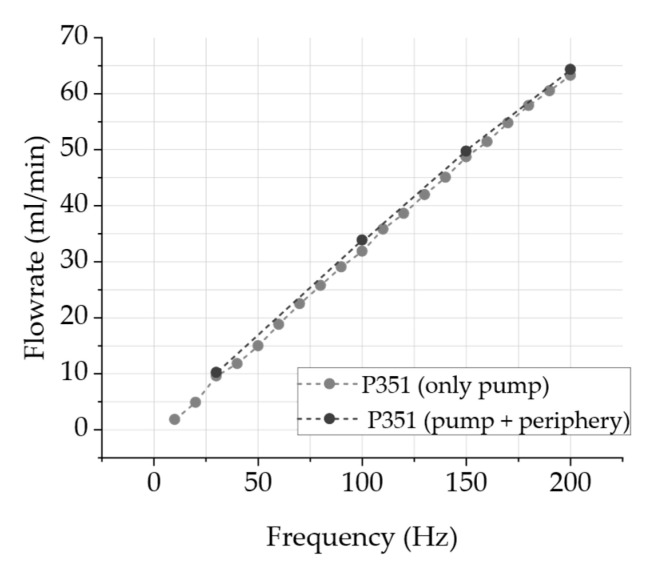
Time-dependent air volume flow with micropump P351 with and without mounted periphery (Figure 3) measured at the outlet with multiple frequencies, rectangular actuation signal and voltages between $U_{-}=-80$ V and $U_{+}=300$ V compared between inlet and outlet.

Figure 7 depicts the comparison between a regularly connected steel micropump without periphery and a fully assembled odor channel. As described in Figure 3, the odor channel consists of a steel micropump, two passive check valves connected in series and an odorant reservoir. The purpose of this measurement is to determine whether these components influence the flow rate. The observed flow rates are almost identical and not significantly affected due to the additional components. The visible deviation is probably a measurement error of the mass flow sensors.

### 3.4. HMD Setup

**Figure 8:**
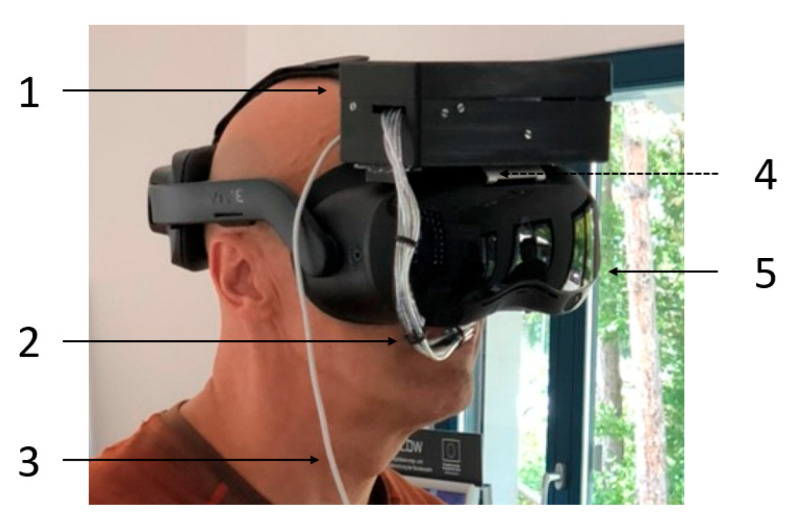
Head-mounted device including the developed prototype (1) of the odor delivery system with micropumps and the VR headset (5), which is attached to an aluminum plate (4). Odor/air mixtures are transported close to the human nose via PFTE tubes (2). The prototype version shown in this figure uses a cable connection (3) for power supply and communication.

The head-mounted device consists of a VR headset (5 in Figure 8) and a prototype of the odor delivery system (1). The prototype is capable of dosing four different odors. It consists of four stainless steel micropumps from Fraunhofer EMFT (Figure 1), each of which is connected to an individual odor reservoir. The inlet and outlet of the reservoirs are sealed with passive check valves (Infuvalve, B. Braun [39]). The odor/air mixtures are transported to the nose through PTFE tubes (2). They are attached to a gooseneck, allowing for optimal positioning directly beneath the nose. Within the housing, the electronics for controlling the micropumps are also integrated. For this prototype, both powering and communication are performed by cable (3). The latest version of the odor delivery system is equipped with wireless connectivity and an internal battery, eliminating the need for external cables. The positioning of the odor delivery system on top of the VR headset was selected to minimize the tubing length and thus the associated dead volume. Mounting to the VR headset was designed with special attention given to the so-called quick release, which allows the system to be quickly fastened to the headset. This was completed by permanently attaching an aluminum plate (4) to the headset and utilizing hook-and-loop fasteners to easily mount the odor delivery system onto the plate. To achieve a lightweight design with a primary focus on wearability, the housing was produced from PLA using fused deposition modeling. The design of the odor reservoirs is aimed at preventing the uncontrolled spread of odors, e.g., through diffusion. For this reason, they are made of epoxy resin using stereolithography and combined with passive check valves to prevent the spreading.

### 3.5. Communication and VR Implementation

**Figure 9:**
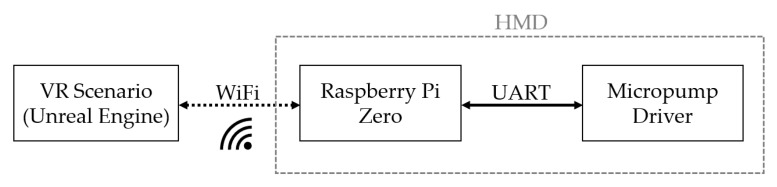
Communication interfaces linking the Unreal Engine VR scenario of a stationary computer with the head-mounted device (HMD) integrated with Raspberry Pi and odor micropumps. The trigger signals from the VR scenario are transmitted wirelessly over WiFi to a Raspberry Pi. The Raspberry Pi subsequently transfers this signal by wire to the micropump driver using the UART protocol.

The VR scenarios are run on a stationary PC, and the data transfer to the VR headset for auditory and visual data is wireless. To ensure a comfortable experience when wearing the odor-dosing unit, the existing WiFi is used to trigger the micropump. A Raspberry Pi Zero (Raspberry Pi Foundation, Model: Raspberry Pi Zero WH) including a battery is integrated inside the odor dosing system to ensure the power supply and enable wireless communication with the micropump driver. Figure 9 shows the detailed sequence of the communication interfaces of the different systems. The communication is established using a TCP socket, ensuring reliable and efficient data exchange essential for synchronizing odor emission with VR stimuli. The Raspberry Pi, equipped with Wi-Fi capabilities, hosts the TCP server. This server listens for incoming connections from the PC and processes commands to trigger the micropump driver. The setup involves configuring the Raspberry Pi to create a socket, bind it to a specific port, and listen for connection requests.

On the PC side, a client application is developed to connect to the Raspberry Pi’s server. Once the connection is established, the PC sends control signals to the Raspberry Pi, instructing it to activate specific micropumps at precise times and varying frequencies, aligning with the VR environment’s sensory requirements. The Raspberry Pi communicates via cable over the UART protocol with the micropump driver, which generates the required high voltage to operate the micropumps and eventually activates the pumps.

**Figure 10:**
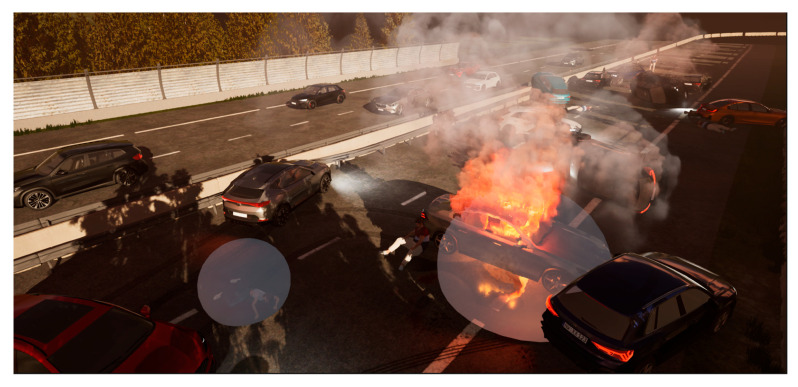
VR scenario implemented in Unreal Engine (© Thera Bytes). Circular gray areas (bubbles) automatically control the fragrance pumps via the communication interface, as presented in Figure 9.

Figure 10 depicts an exemplary VR scenario, created with the software Unreal Engine 5. The scenario involves an accident on a highway with several vehicles involved. One of the cars caught fire, and heavy smoke is visible. Two people are injured on the road, and the emergency personnel must perform the appropriate rescue procedures. In addition, circular gray areas (‘bubbles’) are only visible to the software developers and not to the users. These bubbles with configurable sizes can be placed anywhere in the scenario, typically located around an injured person or in places where the predefined odors match. By entering a bubble in virtual reality, the odor micropump is automatically activated. Each bubble is individually configurable, allowing it to be assigned to an odorant as well as a start and end frequency of the odor micropump. As shown in Figure 4, a higher frequency corresponds to a higher gas flow rate of the micropump, and thus, more odor molecules are delivered towards the nose. Linear and non-linear frequency progressions between the start and end frequencies can be defined through various functions. The start frequency is defined at the outer edge of the bubble, and the end frequency is located in the middle of the bubble. The distance between the HMD and the center of the bubble is measured continuously.

## 4. Discussion

The detailed conceptual description of the planned VR stress training for emergency personnel with visual, acoustic and olfactory stimuli enables the scientific exchange of different research groups involved in the development of virtual stress training scenarios. This promotes a standardized experimental approach and thus supports the comparability of future studies.

The results of the fluidic system validation indicate that the micropump is suitable for the presentation of odors close to the nose with constant amounts of odor-enriched air presentation for varying durations. In detail, the validation of the piezoelectric micropump indicated that the air volume flow of the measured micropumps shows an almost linear behavior of the flow rate with increasing frequency mostly unaffected by the peripheral system of odor reservoir and passive check valves. Hence, we therefore expect to be able to increase or decrease the odor intensity with high precision by adjusting the pump frequency. In addition, we were able to demonstrate that the flow rate remains steady over time at a constant frequency, which is a requirement for a consistent fragrance impression. The use of passive check valves reduces the leakage rate significantly and therefore prevents the uncontrolled outflow of the odorant, as the leakage rate measurement indicates. The measurements of the volume flow between the inlet and outlet of the system show a time delay, which can be defined as the switching time between switching the system on and off as well as between the channels. The time difference until reaching a significant volume flow is in the range of a few seconds and thus within the acceptable range for a congruent perception, according to Murray et al. [12], between visual and olfactory stimuli. This time delay may be reduced in the future with a reasonable effort through a reduction in the dead volume.

The concentration of the inhaled air/odor mixture is a significant factor in the subject’s perception and is highly dependent on the position of the outlet tubes from the odor delivery system. The overall system is lightweight, small, integrated into the VR headset and does not affect the subject’s movements. In addition, the system is wireless and triggered directly from the VR environment, providing a high degree of freedom for customization and calibration between different micropumps.

In comparison to previous studies, the present microdosing system uses valves and should therefore be tighter regarding uncontrolled leakage than systems without valves introduced in former studies. Moreover, the current system can provide four odors at the same time, whereas some earlier systems could only offer one odor (e.g., Guimarães et al. [8]). It is also possible to switch between different odors with this system without the risk of mixing odors. In contrast to the presented olfactory display from Liu et al. [9], no heater is necessary to enable an odor release, so we expect a faster response time and fewer remaining scent molecules to remain perceptible after actuation. This reduces the risk of mixed odors, an issue we are determined to prevent under all conditions.

The development of the microdosing system and its application for civilian stress training in VR has several implications. In contrast to room diffusers of odors, the developed system allows personalized odor presentation in VR. This enables multi-user scenarios in which several emergency personnel undergo stress training at the same time, whereby different odor stimuli can be released depending on a person’s location. Moreover, a personalized odor presentation is of particular interest, as there are large inter-individual differences in odor perception. For example, odors are perceived more intensely by younger people than by older people; women tend to smell slightly better than men, regardless of the odor presented; and in general, smokers have poorer odor performance than non-smokers. Consequently, in addition to the predictor of olfactory stimuli, numerous moderators determine a person’s subsequent reactions or behavior and the resulting consequences. These moderating variables should, therefore, always be recorded in experimental studies, as they allow individual adaptation of the odor presentation (e.g., regarding the perceived intensity of the odor).

## 5. Conclusions and Outlook

The use of odors in the context of civilian stress training in VR is an almost new and highly relevant field of research. The question of whether odors cause stress or whether stress increases the perception of odors is also currently unresolved. One approach is, therefore, to investigate the effects of odors in VR by collecting and evaluating psychological and biophysiological data, as described in the current paper. Machine learning can be a useful approach here to generate new insights from the data.

The fragrance dosing system presented in this paper still has some limitations. When active, the micropump produces an acoustic noise, which participants could interpret as a cue for the subsequent odor presentation. The sound intensity correlates with the activation signal waveform and frequency. To avoid this effect, it is recommended to use the micropump with a sinusoidal waveform combined with an acoustic masking that is played via headphones, which specifically masks ambient noise. The headphones could play brown noise or acoustics that are congruent with the visual input of the VR scenario. In addition, the current microdosing system allows for the simultaneous presentation of four odors. It would certainly be possible to expand the number of odor reservoirs, but this would require reducing the size of the odor reservoirs to ensure that the overall size of the system remains compatible with the HMD together with an adaption of the driver electronics. In conclusion, the current microdosing system is fully functional from a technological point of view. However, independent proband tests to confirm the olfactory function are still pending.

The next steps of the current research project involve testing the developed odor dosing system for the release of odors. This verification will be carried out with proton-transfer-reaction mass spectrometry (PTR-MS). The number of fragrance molecules in the generated volume flow can be precisely detected and compared with human perception. In addition, the reproducibility of the dosed odor quantity, the residence time in proximity to the nose, and the response time should be demonstrated and verified. Furthermore, the number of odor channels can be significantly increased through miniaturization, allowing even more sophisticated experiences. Then, the system could be placed closer to the subject’s nose, as the switching time and delay are reduced by decreasing the dead volume of the tubing. A further step is to conduct a participant study to determine the intensity, pleasantness, subjective physiological arousal and familiarity of selected odors as perceived by individuals. These odors are then tested again in the two VR scenarios to determine interactions between visual, acoustic and olfactory stimuli regarding (potentially interindividual different) psychophysiological effects for emergency personnel. The results will help to improve the training by preparing emergency personnel for deployment in a more realistic way.

The VR worlds will be completed and evaluated. Scenario 1 will be evaluated using emergency personnel. One focus of this evaluation is on the effects of VR training on the resilience of emergency personnel and how it differs from conventional training methods, with special consideration of the effects of auditory, visual and olfactory stressors. The overarching question is: Can stress be generated in VR? And can suitable methods for stress reduction be taught in VR? We also take an idiographic approach: what works best for whom, under which conditions, and why? Scenarios 2 and 3 will be used in the training of emergency personnel from next year by the “Bayerisches Zentrum für besondere Einsatzlagen” (Bavarian Center for special situations: Simulation Center for Medical staff in life-threatening situations). With the planned studies, we will be able to present olfactory stimuli in psychophysiological stress training in VR to improve perceived realism in current training. The aim of further research is to find out to what extent the integration of odors can improve the training of emergency personnel.

## 6. Patents

1. Linssen R.; Richter, M.; Kibler, S. Controllable Scent Sample Dispenser—European Patent Office—EP 2706838 B1: Steuerbarer Duftprobenspender [Online]. 9 November 2016.
2. Richter, M.; Kibler, S.; Kruckow, J. Controllable Fluid Sample Dispenser and Methods Using the Same—European Patent Office—EP 2707066 B1: Steuerbarer Fluidprobenspender und Verfahren zur Verwendung Desselben [Online]. 16 December 2015.

## Data Availability

Measurement data are available upon request from the first authors.

## References

[1] Howell M.J., Herrera N.S., Moore A.G., McMahan R.P. (2016). A reproducible olfactory display for exploring olfaction in immersive media experiences. Multimed. Tools Appl..

[2] Herman S., Barnum T.C., Minà P.E., Wozniak P., van Gelder J.-L. (2024). Affect, emotions, and crime decision-making: Emerging insights from immersive 360° video experiments. J. Exp. Criminol..

[3] Sona B., Dietl E., Steidle A. (2019). Recovery in sensory-enriched break environments: Integrating vision, sound and scent into simulated indoor and outdoor environments. Ergonomics.

[4] Baus O., Bouchard S. (2017). Exposure to an unpleasant odour increases the sense of Presence in virtual reality. Virtual Real..

[5] Baus O., Bouchard S., Nolet K., Berthiaume M. (2022). In a dirty virtual room: Exposure to an unpleasant odor increases the senses of presence, reality, and realism. Cogent Psychol..

[6] Patnaik B., Batch A., Elmqvist N. (2019). Information Olfactation: Harnessing Scent to Convey Data. IEEE Trans. Visual. Comput. Graphics.

[7] Tewell J., Ranasinghe N. (2024). A Review of Olfactory Display Designs for Virtual Reality Environments. ACM Comput. Surv..

[8] De Paiva Guimarães M., Martins J.M., Dias D.R.C., Guimarães R.d.F.R., Gnecco B.B. (2022). An olfactory display for virtual reality glasses. Multimed. Syst..

[9] Liu Y., Yiu C.K., Zhao Z., Park W., Shi R., Huang X., Zeng Y., Wang K., Wong T.H., Jia S. (2023). Soft, miniaturized, wireless olfactory interface for virtual reality. Nat. Commun..

[10] Niedenthal S., Fredborg W., Lundén P., Ehrndal M., Olofsson J.K. (2023). A graspable olfactory display for virtual reality. Int. J. Hum.-Comput. Stud..

[11] Richter M. (2017). Microdosing of Scent-Handbook of Odor.

[12] Murray N., Qiao Y., Lee B., Muntean G.-M. (2014). User-profile-based perceived olfactory and visual media synchronization. ACM Trans. Multimedia Comput. Commun. Appl..

[13] Bußmann A.B., Durasiewicz C.P., Kibler S.H.A., Wald C.K. (2021). Piezoelectric titanium based microfluidic pump and valves for implantable medical applications. Sens. Actuators A Phys..

[14] Herz M., Wackerle M., Bucher M., Horsch D., Lass J., Lang M., Richter M. (2008). A Novel High Performance Micropump for Medical Applications. https://www.researchgate.net/profile/M-Richter/publication/267550233_A_Novel_High_Performance_Micropump_for_Medical_Applications/links/54cf37610cf24601c0930f48/A-Novel-High-Performance-Micropump-for-Medical-Applications.pdf.

[15] Zengerle R., Ulrich J., Kluge S., Richter M., Richter A. (1995). A bidirectional silicon micropump. Sens. Actuators A Phys..

[16] Cantwell M.L., Amirouche F., Citerin J. (2011). Low-cost high performance disposable micropump for fluidic delivery applications. Sens. Actuators A Phys..

[17] Streb M., Michael T. (2015). Posttraumatische Belastungsstörung bei medizinischen Rettungskräften. PiD—Psychother. Dialog.

[18] Ein N., Plouffe R.A., Liu J.J.W., Gervasio J., Baker C., Carleton R.N., Bartels S.A., Lee J.E.C., Nazarov A., Richardson J.D. (2024). Physical and psychological challenges faced by military, medical and public safety personnel relief workers supporting natural disaster operations: A systematic review. Curr. Psychol..

[19] Marmar C.R., Weiss D.S., Metzler T.J., Ronfeldt H.M., Foreman C. (1996). Stress responses of emergency services personnel to the loma prieta earthquake interstate 880 freeway collapse and control traumatic incidents. J. Trauma. Stress.

[20] Raphael B., Singh B., Bradbury L., Lambert F. (1984). Who Helps the Helpers? The Effects of a Disaster on the Rescue Workers. Omega.

[21] Dalton P. (2004). Odors, Deployment Stress, and Health: A Conditioning Analysis of Gulf War Syndrome.

[22] Howard M.C., Gutworth M.B., Jacobs R.R. (2021). A meta-analysis of virtual reality training programs. Comput. Hum. Behav..

[23] Dinh H.Q., Walker N., Hodges L.F., Song C., Kobayashi A. (1999). Evaluating the importance of multi-sensory input on memory and the sense of presence in virtual environments. Proceedings of the IEEE Virtual Reality (Cat. No. 99CB36316), Virtual Reality.

[24] Ghinea G., Ademoye O. (2012). The sweet smell of success. ACM Trans. Multimedia Comput. Commun. Appl..

[25] Archer N.S., Bluff A., Eddy A., Nikhil C.K., Hazell N., Frank D., Johnston A. (2022). Odour enhances the sense of presence in a virtual reality environment. PLoS ONE.

[26] James K.A., Stromin J.I., Steenkamp N., Combrinck M.I. (2023). Understanding the relationships between physiological and psychosocial stress, cortisol and cognition. Front. Endocrinol..

[27] Van Dammen L., Finseth T.T., McCurdy B.H., Barnett N.P., Conrady R.A., Leach A.G., Deick A.F., van Steenis A.L., Gardner R., Smith B.L. (2022). Evoking stress reactivity in virtual reality: A systematic review and meta-analysis. Neurosci. Biobehav. Rev..

[28] Finseth T.T., Smith B., van Steenis A.L., Glahn D.C., Johnson M., Ruttle P., Shirtcliff B.A., Shirtcliff E.A. (2024). When virtual reality becomes psychoneuroendocrine reality: A stress (or) review. Psychoneuroendocrinology.

[29] Giannakakis G., Grigoriadis D., Giannakaki K., Simantiraki O., Roniotis A., Tsiknakis M. (2022). Review on Psychological Stress Detection Using Biosignals. IEEE Trans. Affect. Comput..

[30] Lu L., Zhu T., Morelli D., Creagh A., Liu Z., Yang J., Liu F., Zhang Y.-T., Clifton D.A. (2024). Uncertainties in the Analysis of Heart Rate Variability: A Systematic Review. IEEE Rev. Biomed. Eng..

[31] McAllister M.J., Martaindale M.H., Gonzalez A.E., Case M.J. (2022). Virtual Reality Based Active Shooter Training Drill Increases Salivary and Subjective Markers of Stress. Yale J. Biol. Med..

[32] Mentis A.-F.A., Lee D., Roussos P. (2023). Applications of artificial intelligence-machine learning for detection of stress: A critical overview. Mol. Psychiatry.

[33] Kim H., Kim D.J., Kim S., Chung W.H., Park K.-A., Kim J.D.K., Kim D., Kim M.J., Kim K., Jeon H.J. (2021). Effect of Virtual Reality on Stress Reduction and Change of Physiological Parameters Including Heart Rate Variability in People With High Stress: An Open Randomized Crossover Trial. Front. Psychiatry.

[34] Santos D.V., Reiter E.R., DiNardo L.J., Costanzo R.M. (2004). Hazardous events associated with impaired olfactory function. Arch. Otolaryngol. Head Neck Surg..

[35] Daniels J.K., Vermetten E. (2016). Odor-induced recall of emotional memories in PTSD-Review and new paradigm for research. Exp. Neurol..

[36] Loos H.M., Schreiner L., Karacan B. (2020). A systematic review of physiological responses to odours with a focus on current methods used in event-related study designs. Int. J. Psychophysiol..

[37] Höferl M., Krist S., Buchbauer G. (2006). Chirality influences the effects of linalool on physiological parameters of stress. Planta Med..

[38] Springer A., Höckmeier L., Schicker D., Hettwer S., Freiherr J. (2022). Measurement of Stress Relief during Scented Cosmetic Product Application Using a Mood Questionnaire, Stress Hormone Levels and Brain Activation. Cosmetics.

[39] B. Braun Melsungen AG (2024). Infuvalve—Back-Check Valves: B. Braun′s Only Drug Resistant Back-Check Valve.

[40] Benedek M., Kaernbach C. (2010). A continuous measure of phasic electrodermal activity. J. Neurosci. Methods.

[41] Braithwaite J.J., Watson D.G., Jones R., Rowe M. (2013). A guide for analysing electrodermal activity (EDA) & skin conductance responses (SCRs) for psychological experiments. Psychophysiology.

[42] Topoglu Y., Watson J., Suri R., Ayaz H., Ayaz H. (2020). Electrodermal Activity in Ambulatory Settings: A Narrative Review of Literature. Advances in Neuroergonomics and Cognitive Engineering.

[43] Makowski D., Pham T., Lau Z.J., Brammer J.C., Lespinasse F., Pham H., Schölzel C., Chen S.H.A. (2021). NeuroKit2: A Python toolbox for neurophysiological signal processing. Behav. Res. Methods.

